# Some Reflections on Research in Medicine
*An address delivered to the "Galenicals" in the University of Bristol on Tuesday, 4th May, 1937.


**Published:** 1937

**Authors:** F. J. Poynton

**Affiliations:** Consulting Physician to University College Hospital; to Great Ormond Street Children's Hospital; and to Winford Orthopædic Hospital


### SOME REFLECTIONS ON RESEARCH IN MEDICINE.*

BY

F. J. Poynton, M.D., F.R.C.P.,
*Consulting Physician to University College Hospital; to Great Ormond Street Children's Hospital; and to Winford Orthopædic Hospital.*

---

Ladies and Gentlemen, for my own sake and for the sake of medicine I hope you are not all Galens, for an intellect such as his was so commanding that eventually its unquestioned authority tended to become an obstacle to progress rather than to assist its advance.

I have chosen "Reflections on Research" for my subject, and shall from time to time illustrate it from the ancient history of rheumatism. But be under no mistake. I am not supposing or wishing that you should all become what are sometimes called "Researchers." A great part of my professional life was spent teaching general medicine to students with a view to the most important task of general practice. The great work of doctors is done in ordinary general practice, not in whole-time research; but it is, I think, a great advantage to preserve some of the characteristics of the research mind, whatever line you may take up. These prevent one from getting too much in a groove, and from becoming that rather dreadful person a

* An address delivered to the "Galenicals" in the University of Bristol on Tuesday, 4th May, 1937.

self-made authority on a subject upon which the wisest doctors only know surprisingly little.

It must have struck you how strange it is that when there is a difficult case frequently an able man of many years' experience is baffled as much as you are yourselves: that is supposing you have escaped from that first evidence of ignorance which leads you when a clinical clerk of two months' standing to believe that you know more than the experienced. Even a resident may be a very trying individual with his air of importance, and I speak from a long experience of residents, mostly good, some moderate, a few definitely bad. When I analyse my bad ones they have been either lazy or have continually insisted upon teaching me. I say *continually*, for there is no sensible physician who cannot learn something from a younger mind.

There are very different ideas about research, and the word "genius" frequently crops up over this pursuit. I do not attempt to define genius. It is most commonly discovered, we know, after the individual is dead, but I will give you my idea of genius. A man watches a small boy blowing a fine soap bubble, and argues from this that the soapy medium forms the envelope of that bubble and that the heated air inside causes it to float. He takes a small paper bag, fills it with hot air, ties the neck and sees it rise in the air. He perseveres and makes a balloon 35 feet high, 110 feet in circumference, weighing 500 lbs., and demonstrates that it also will ascend in the air. Then he notices that before it is full it pulls so hard to ascend that it requires men and ropes to hold it *down*. Accordingly he argues it will take something *up*, and eventually sees human beings riding through the air and has laid the foundation of aeronautics.

He has shown much more than "the infinite capacity for taking pains," at the best only one feature of genius. He has seen what we did not see, he has visualized what might possibly result from his observation, and turning neither to the right nor left has, step by step, rationally developed his discovery. Most geniuses are simple-minded, because every step of that epoch-making discovery was simple when you come to think of it to-day and to look back on it.

Research in medicine is vastly more difficult because of the great complexity of human life and disease, but the general principles are the same—some little secret missed for years suddenly explained, some scattered areas of knowledge drifting along by themselves suddenly linked together by a penetrating mind, some new discovery not directly connected with medicine suddenly recognized as a starting-point for a new medical development.

Rheumatism was thought by the ancients to be a peccant humour or rheum spreading down from the brain, but Radulfe in the thirteenth century focused his attention on the joints and muscles and spoke of "gout," which Aretæus in the second century had with some truth observed none but the gods understood. The peccant humour or rheum had now, you see, become something wrong with the joints. Then Ballonius in the seventeenth century noticed that not all arthritis was gouty, and called these other acute forms "rheumatism."

Then old bones were dug up which showed that arthritis was not always acute but was sometimes very chronic, and this led to Sydenham trying to evade this difficulty by calling this chronic form "scorbutical rheumatism." Next Störck started a new line of thought, for by post-mortem examinations he showed in 1762 that rheumatism was not only an arthritis but that there were visceral complications. Then during the French Revolution Beauvais recognized what we call rheumatoid arthritis from among the more chronic forms of arthritis.

In 1789 Edward Jenner—a general practitioner, mark you — not content only with vaccination, recorded heart disease and rheumatism. Then came the genius of Lænnec with the stethoscope in 1819, and in 1836 the great French physician Bouillard, by establishing the association of acute carditis with rheumatism, left in continental literature the term Bouillard's disease for acute rheumatism of childhood.

Sydenham, writing on chorea, and a little earlier Stoll in 1789, began to show us its association with rheumatism, and Begbie in 1847 maintained it was not due to heart disease, but with heart disease was also caused by rheumatism.

Another French genius arrived named Pasteur, and for nearly forty years the idea that some rheumatism, especially children's rheumatism, might be due to infection was being worked at all over the world. Yet do not think we are more than on the fringe of understanding "rheumatism": indeed I sometimes think the ancient "rheum" or "peccant humour" has ceased flowing down from the brain of the rheumatic, and is now flowing down from the brains of all of us who are fighting over what is meant by rheumatism and what are its causes.

In spite of this pathetic conclusion, I cannot help feeling that all of you who have listened to these stories of the air balloon and of rheumatism must feel a sympathetic chord has been touched with our modern literature, the Penguin books, price 6d., in which you trace the activities of the detective or a rival amateur genius discovering the murderer.

When you are studying medicine to pass examinations — an absolute necessity — you are naturally concerned with what are looked upon at the moment as facts. You must have facts to achieve success in an examination, and you must pass examinations, but a fact is the end of its research. When I say a to-and-fro murmur heard over the aortic cartilage is evidence of aortic regurgitation, and prove it to you by a post-mortem examination, the research on that particular point is at an end, for it has become a fact. The " cram " books on medicine—mind, I do not decry them—largely consist in a collection of bottled facts, and one great objection to repeated examinations is the danger that your mind is deadened by learning facts, that is you are continually reaching full stops.

Perpetual teaching, or " spoon feeding " as it is called, from the earliest moments in the wards, does not assist the research spirit. Now if you blunder through the examination of a case and present your view of it to me, naturally enough I might say I think you have missed the point here, this is the point you want. But you have, nevertheless, undertaken a research, and like everyone beginning to research and even ending research you may have missed the essential, but you have strengthened your intelligence, not dulled it or kept it stationary.

There is a curious idea that someone who drifts into a laboratory to research is a superior being. He may or may not be. He may carry on some work already outlined but needing to be studied in more detail. That is good work, but it is not superior to the work of someone in the wards confirming some new clinical observation already made but requiring more confirmation. This is often called "hack work." I don't call it hack work, but useful work, though not mysterious or marvellous work. This, then, is no superior being because he is in a laboratory, he is not more marvellous than his friend the clinician he laughs at.

It is clearly, then, most important that we recognize that there is clinical research and laboratory research, and post-mortem evidence too when that is needed to keep us steady. There is no place on either side for antagonism. It must be remembered that clinical research may anticipate laboratory research, although the exact explanation of the clinical observation may have to await the laboratory investigator.

I knew the great children's physician, Dr. Cheadle, very well, and he told me that as a result of his travels in Canada he had seen and learnt much about scurvy in adults, and that on returning to England he found doctors terribly puzzled over a condition in infants brought about by patent foods. No one knew how to treat it or what it really was in nature. He from his observations and research into the scurvy of adults at once realized it to be scurvy in infancy, and he cured it by fresh foods.

He insisted that there was a lack of something which he called the antiscorbutic element in the patent foods, because he had studied adult scurvy and its causation. Now you all recognize that element as Vitamin C, but many infants had been saved before you knew this or I knew it, and one of the duties of a doctor is to save life, even if he cannot give the name Vitamin C.

The reverse, of course, occurs. I have only to mention insulin and many other instances will occur to you.

I am putting aside for simplicity's sake any question of the financial side of our subject, though I would remind you that the great investigators have often worked in very small laboratories, and that a magnificent laboratory does not always mean good work, though it is a great asset to good work.

It is useless to lay down laws for the born investigator. He or she stands apart from us, with that curious gift of seeing something that seems new; that outstanding patience against failure or derision, because he knows that it is something new, and that concentration on the goal ahead. He may not succeed or he may be wrong, but he leaves a track of some kind which is of value, and by his mental power trains others who may surpass him in results. Such minds are invariably stimulating in the boldness of their outlook and tenacity of purpose, but they are minds which obey their own laws.

Now when the laboratory investigator, as a research worker, begins to scoff at and deride the clinician he is in a bad way. I have been a pathologist, a bacteriologist, and a clinician in my time, and I have worked shoulder to shoulder with each type, though I became a clinician eventually because I loved the human side. I admire all types, but when a vaccine specialist living in a laboratory undertakes to cure a case of malignant endocarditis, and after his injections the temperature slowly falls and the patient dies, and he does not come to the post-mortem examination; further, when he is content to say if he had commenced the treatment sooner the patient would have lived, I think badly of him. Why ? Because he did not know what the clinician knows, that the fall in temperature was the sign that the patient's resistance was exhausted, and not of the partial success of a vaccine. If he had come to the autopsy he would have seen the impossibility of his claim, but as it is he returns to his laboratory believing in a partial success, and he is in a bad way.

In this case you see two instances of research, the laboratory research which I do not criticize, and the clinical research which led the physician to recognize from his knowledge of malignant endocarditis and from his knowledge of his patient that the fall of temperature was premonitory to death. The weak point was the ultimate failure in research of the laboratory investigator in not facing the findings of the post-mortem examination. The physician could also have told him that he had seen vaccines used at the earliest moment of detection of malignant endocarditis and had even then seen them fail.

If, however, a physician denied the possibility that at some future time a vaccine might be discovered which would be successful, he would be speaking without the requisite knowledge of the possibilities of laboratory research, and would be also moving on bad lines, because he could not possibly know the future of laboratory research.

Take another case. A physician has under his care a case of anæmia which he *assumes* can be cured with massive doses of iron, but the case does not improve. He is persuaded to call in from the laboratory an expert upon examination of the blood, who at once points out that it is a pernicious anæmia. Here the physician has failed in his research mind, and has assumed that he knew from his own experience something that the laboratory research worker could prove at once to be an erroneous assumption from his laboratory researches.

To recapitulate at this point. I have been laying stress on the belief that the essentials of the research mind are to be found in wards, at the bedside, as well as in laboratories; that the various research minds should try to keep in touch with the main object—the advancement of medicine—and not become aloof from one another; and that the outstanding research mind is one which makes its own laws and is not guided by ours; and, lastly, that such minds have a stimulating effect upon us by their peculiar quality and power to make us think.

You may ask: How can the research mind help in every-day work? It can help from the first. For instance, in reminding you that you are not studying a pneumonia, but a case of pneumonia, a human being suffering from pneumonia. Then you begin to realize the truth, for you find yourself inquiring in your mind, why did this case do well and that one do badly ? You are led to research away from the pneumonia into the type of pneumonia, into the influence of age, of alcoholism, of overwork and under-nourishment and so on. It is this habit of mind that results, after the necessary experience brought alone by time, in making the competent doctor, the practitioner who becomes thoroughly trusted because his judgement is sound. Year after year, unconsciously perhaps, he has been researching not only into pneumonia, but into how pneumonia and other illnesses may affect this or that individual.

I could see the type of mind when I was examining. When, for example, I asked some question upon treatment, and the answer came, "If I had seen this case earlier I should have advised immediate operation, but I am not certain that now the risk will not be too great." The particular condition under consideration was the same from the first, but the examinee had recognized that the human being had altered : he had become too weak, or too old, or some complication had arisen making the issue doubtful. In this answer I recognized the research mind. There is no doubt that when a student the rather wearisome undertaking of noting down each day some curious point, some interesting comment, or some unexpected happening about one's cases assists the research mind. When qualified and in practice you may laugh over your old notes as I have over mine, but they served their purpose.

We must realize that the research mind is to some extent adventurous and takes the risks of adventure, and to me it has seemed that when a particular physician asks questions of students that those who keep well in the back row to avoid answering them lose thereby.

You may be quite sure of this. If you eventually do some research, so often called original—but so rarely as you will be surprised to find actually original—you will have to face up to criticism and to cultivate a placid, I don't say a pacific, mind to carry you along. If the work is important and you believe sound certainly you must defend it, but if from studentship you have feared criticism and making some mistakes you will not easily keep your equanimity later, and you may even turn tail.

There is much to be learnt, of course, in laboratory training just as there is in surgery—much accuracy, and in some cases much knowledge of handling instruments, and so on, before the actual investigation can be safely undertaken. Some minds naturally enough prefer the wider, more philosophical nature of medicine, or the practical details of preventive medicine, but they are not safe from research.

You never know, in whatever branch of the profession you are, whether opportunities may not arise which put you into the position of making some useful contribution. This leads me to another consideration, the art of putting your work into published form. This is also a type of research. It is a research into the minds of those whom you think will read your work. Do not suppose I am going to advise you to study my writings for this purpose. I have written three books, numerous lectures and a dreadful number of medical articles, and I was told recently by the candid friend and the gentle critic that

I wrote "journalese." I am by nature not easily moved, and love the humorous side of things, and my retort was an easy one. It was: "When you have three books published and are asked for—I do not mean send—reprints of your lectures come and see me again and talk it over."

There are great pitfalls in writing. One is, to suppose the reader knows the subject as well as you do yourself. Another to use a number of long scientific words when only a few are necessary. Another to suppose that complicated graphs in great numbers can appeal to a reader who is not acquainted with complicated diagrams. Another to presume that everyone who may not agree with your new and perhaps debatable conclusions is a fool. Even if I am a fool I do not like to feel it, and the enemy bides his time to strike back.

A summary at the end of your paper should avoid the obvious. Such as this (No. 1): "We have shown that rheumatism is a common disease." We all know that already. Or an ending such as this (No. 2): "We have investigated *x* number of cases for such and such a point, but it will require many more before we can decide upon the real interpretation." (No. 3): "We publish these results as a preliminary step." Why publish them if you want many more cases to prove the meaning of your point? Why not wait and not trouble to inform the medical profession rheumatism is a common disease. I am very suspicious of a preliminary report, because so often one never hears the sequel. It reminds me of showing a difficult clinical case you cannot diagnose and never reporting later the fate of that case. Lastly remember: "What is written is written." One often regrets this. Don't be surprised if, in spite of accuracy, you may find your view not understood, or garbled. Be patient.

There is another aspect of research, an interesting one. This is the tendency to specialization, and probably in the end complete specialization.

It is a clear impossibility to investigate everything in medicine, but I do think this, that we should try to realize that when we take up special lines of investigation we narrow our outlook on human illness, and may lose a sense of proportion. We should be on guard lest we become fanatics. There are plenty of instances of able people who have found out a half-truth—and it may be a valuable half-truth—and have then surrendered their research mind to fanaticism. More and more is to be explained by this half-truth, until at last everything is to be explained. Such able minds may become actually deranged. Doctors who have to teach students are much protected against this, for the student requires varied teaching. Again, those who can listen to other views are much protected. You can quite easily become a fanatic over rheumatism. You can become a fanatic over the bacteriology, the endocrines, the diet, the gold as a drug or as a reward, the local focus, the rheumatic diathesis, bee venom, the climate, the sympathetic system and allergy.

How can you prevent this danger? I think by not shutting yourself up in the chamber in which your particular idea is living, but by listening to the words of others living in other chambers, perhaps they also inclined to be fanatics. You say to me, "Malfunction of the endocrines explains all rheumatism." And I ask, "Does a child aged three with Still's disease or juvenile rheumatoid arthritis suffer from endocrine malfunction? What is your proof of that?" I say to you, "All rheumatism is due to an infection." You say to me, "What is rheumatism?" Even as Pilate said, "What is truth?" If I am honest I say, "I don't really know." And you reply, "How, then, do you know all rheumatism is due to an infection?"

Signs of fanaticism appear sometimes early in a student's career, and I don't like to see it, because it grows with age. Fanatics, it is true, have done wonders in great causes, but I think in the complicated study of human disease it is more frequently the mind that is balanced, yet has some great truth to make known, that is the more often the one that obtains far-reaching results, because it knows that this truth cannot be forced upon unwilling ears, but must wait for results which bring conviction. Lister held firmly to the great principle of antisepsis, and he won his way by continuous efforts to show that the results justified his faith, not as a fanatic, but as a man who saw that the truth must justify itself and not be thrust against the unwilling, thereby increasing resistance to its acceptance.

One of the great difficulties in research is the doubt as to whether a result is of any use. We must admit that a great deal of research seems to be lost, but we have also to admit that we never know when something apparently lost and useless may not be found to be of value. Many a man has suffered the fate during life of finding all his work apparently useless. Later, when he is dead, some change in thought has occurred and the true value of his work is suddenly discovered. I do not think anyone can guard against this. It is the fate that comes to pioneers in all departments of life, and the only consolations are the pleasure and interest of the work to the individual and the grimmer joy of combating the adversary.

Some of you may not realize how long a time it takes to get a grasp of the subject you wish to investigate. Rheumatism, for example, requires at least two years' study before you can grasp the difficulties and the problems. Yet there is a tendency to give short time aids to research on such a subject to a beginner. It seems to me that the time is up before he has had a chance to know the outlines of the subject, and yet he is obliged, perhaps, to produce some result, and produces through no real fault a very imperfect one. Then, maybe, he considers he has spent enough of his life on this investigation and he gives it up. A good deal of money is easily wasted upon research, as you can guess. It is inevitable.

It is a very interesting point for the philosopher to consider whether ground which has been already explored and supposed to have yielded all its usefulness should be again explored by a beginner. If it is very important ground I think yes, for though you may arrive at the same results, you may see things from a different angle to the forerunner, and as a result side tracks be started from the main track.

Some things are so well established, so certain, and so thoroughly recognized that continual repetition is not helpful. They have become facts, and most important ones, but not requiring continual repetition, unless there are some side tracks to be learnt from them. There are limits to repetition, especially when there is so much that is new to discover.

We cannot escape from the fact that some minds are not meant for research in laboratories, though the individuals may believe they have this flair. Soon they get into a groove and lose that "vision" which is required, and as you will know when you get to middle age, though some outstanding minds do not weaken in vision, most, like the singer, find themselves driven to sing the old songs that were once attractive but are not new. The mind of a child does not remain with most of us, but it is the mind of a child that is so original, because unfettered by custom it sees very clearly the essential.

